# Leukemia With *TCF3-ZNF384* Rearrangement as a Distinct Subtype of Disease With Distinct Treatments: Perspectives From A Case Report and Literature Review

**DOI:** 10.3389/fonc.2021.709036

**Published:** 2021-07-28

**Authors:** Na Lin, Xiaojing Yan, Dali Cai, Lei Wang

**Affiliations:** Department of Hematology, The First Affiliated Hospital of China Medical University, Shenyang, China

**Keywords:** *TCF3-ZNF384* fusion, acute lymphoblastic leukemia (ALL), mixed phenotype acute leukemia (MPAL), *ZNF384* rearrangement, treatment

## Abstract

**Background:**

*ZNF384* rearrangements are found in 5-10% of B-cell acute lymphoblastic leukemia (B-ALL) and 48% of B cell/myeloid mixed phenotype acute leukemia (B/M MPAL). *ZNF384*-rearranged B-ALL is prone to lineage conversion after chemotherapy. *TCF3* is the second most common rearrangement partner of *ZNF384* in B-ALL (27.5%) and the most common partner in B/M MPAL (53.3%). *TCF3-ZNF384* fusion is related to a poor steroid response and a high frequency of relapse. It is mostly reported in children and adolescents but rarely seen in adults.

**Patients and Methods:**

Here, we report a rare case of adult common B-ALL with *TCF3-ZNF384* fusion in which the patient relapsed after one cycle of consolidation chemotherapy. Relapsed leukemia cells from the bone marrow were cultured for 72 hours ex vivo, and a panel of 156 kinds of cytotoxic drugs, targeted therapy drugs, combination chemotherapy drugs, etc., was used for sensitivity screening. The literature on *TCF3-ZNF384* fusion was reviewed, and reported cases with *TCF3-ZNF384* fusion were summarized. Clinical characteristics were compared between B-ALL and MPAL with *TCF3-ZNF384* fusion.

**Results:**

The relapsed lymphoblasts showed moderate sensitivity to both acute myelocytic leukemia (AML) - and acute lymphoblastic leukemia (ALL)-directed combination chemotherapy schemes, as well as to multiple targeted therapeutic drugs. The hyper-CVAD (B) scheme showed synergistic effects with multiple targeted compounds and had the highest sensitivity. The patient chose the hyper-CVAD (B) scheme combined with sorafenib and achieved complete remission (CR), then consolidated with myeloid-directed homoharringtonine+cytarabine+daunorubicin (HAD) scheme and gained molecular CR. By reviewing the literature, we found that both the genomic landscapes and gene expression profiles of *ZNF384*-rearranged B-ALL and MPAL are similar and that both diseases have lineage plasticity. The gene expression profile in *TCF3-ZNF384*-positive patients shows enrichment of hematopoietic stem cell features. No significant differences in clinical characteristics were found between *TCF3-ZNF384*-positive ALL and MPAL.

**Conclusion:**

*TCF3-ZNF384*-positive leukemia may be a distinct subtype of leukemia regardless of immunophenotype. Considering the frequent lineage switches and sensitivity to both ALL- and AML-directed schemes, a uniform strategy directed at both lymphoid and myeloid lineages or at hematopoietic stem cells may be better for *TCF3-ZNF384*-positive leukemia. Small molecule targeted therapies may be promising treatment options and deserve further investigation.

## Introduction

Cytogenetic and molecular genetic information has great value in the diagnosis, treatment and prognostic evaluation of acute leukemia. For example, patients with the *AML/ETO* fusion are diagnosed with acute myeloid leukemia (AML) with *AML/ETO* regardless of the percentage of leukemia cells and are believed to have a good prognosis (1). *ZNF384* rearrangement is found in 5-10% of B-cell acute lymphoblastic leukemia (B-ALL) (2–5) and 48% of B cell/myeloid mixed phenotype acute leukemia (B/M MPAL) (6). *ZNF384*-rearranged B-ALL often presents as CD10-negative, accompanied by aberrant expression of the myeloid markers CD13 and CD33 (5), and is prone to lineage conversion after chemotherapy. To date, 15 different rearrangement partners of *ZNF384* have been found (7). Among them, *TCF3* is the second most common partner in *BCP-ALL* (27.5%) (7) and the most common partner in B/M MPAL (53.3%) (6). B-ALL patients with *TCF3-ZNF384* fusion have a poor steroid response and a high frequency of relapse (5). This scenario has mostly been reported in children and adolescents but rarely seen in adults. Liu, Y. F. sequenced samples from 177 adult B-ALL patients, and *TCF3-ZNF384* fusion was not detected (4).

Here, we present the treatment process of a case of adult B-ALL with *TCF3-ZNF384* fusion and *in vitro* drug sensitivity screening results. Through this case and literature review, we provide evidence that leukemia with *TCF3-ZNF384* rearrangement is a distinct disease needing distinct treatments, and this idea deserves further investigation.

## Case Presentation

**Figure 1:**
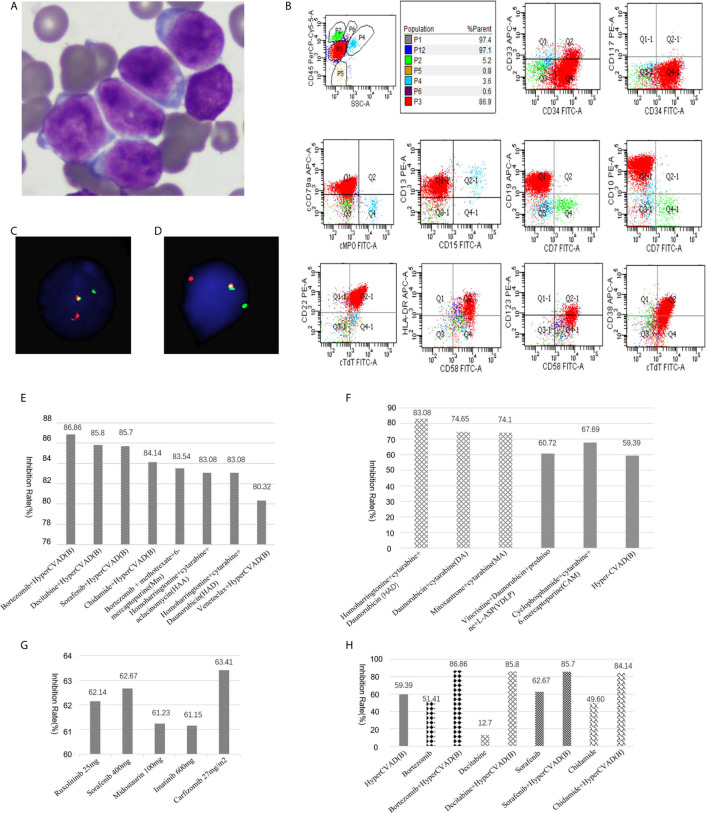
**(A)** Morphology of lymphoblasts at diagnosis (original magnification, × 1000). **(B)** Flow cytometry result. **(C)** FISH results using a break-apart probe for TCF3. Rearrangement of TCF3 was shown as one red and one green signal. **(D)** FISH results using a break-apart probe for ZNF384. **(E)** Drug sensitivity screening test in vitro with a panel of 156 kinds of cytotoxic drugs, molecular targeted therapy drugs, and combination chemotherapy regimens. Relative inhibition rates higher than 80% were listed. **(F)** Relapsed lymphoblasts showed moderate sensitivity to both an ALL scheme [VDLP, CAM, hyper-CVAD **(B)**] and AML schemes (HAD, DA, MA). **(G)** Relapsed lymphoblasts were sensitive to multiple targeted therapeutic drugs. **(H)** The hyper- CVAD **(B)** scheme showed multiple synergistic effects with multiple targeted compounds.

A 41-year-old male was admitted to our hospital in November 2020 due to a fever that had been present for two weeks with a maximum temperature of 38.2°C. He had no relevant genetic family history. Physical examination showed multiple enlarged lymph nodes in the groin and axilla, which were firm and showed moderate activity, with the largest lymph node being approximately 2.5 cm in diameter. The blood tests showed a white blood cell (WBC) count of 150×109/L, a hemoglobin (HGB) level of 101 g/L and a platelet count of 17×109/L. Bone marrow aspiration was performed and revealed hypercellularity with 98.4% lymphoblasts. He was diagnosed as L2-type ALL according to FAB classification (**Figure 1A**). Flow cytometry showed malignant B lymphoblasts (P3 group, 92.1%) mainly positive for CD34, CD19, CD10, cCD79a, TdT, CD22, HLA-DR, CD58, and CD13; partially positive for CD38 and CD123; weakly positive for CD33; and negative for CD117, CD7, CD3, MPO, cCD3, CD56, CD15, CD79a, cIgM, CD25, CD20, surface Kappa and Lambda, in accordance with com-B-ALL (**Figure 1B**) (Experiment was conducted by CANTO II, 6 color flow cytometry, Becton, Dickinson and Company(BD) and all antibodies were purchased from BD company). Karyotype analysis showed that the patient had a 46,XY karyotype [20]. Quantitative polymerase chain reaction (qPCR) covering 56 commonly detected fusion genes in leukemia (listed in **Supplementary Table 1**) detected only the expression of WT1. Next generation RNA sequencing of bone marrow cells detected a rare fusion (*TCF3-ZNF384*), a *FLT3* mutation c.2028C>A|p.Asn676Ly), a *TCF3* mutation (c.1061delC|p.Ser354fs), a *NOTCH2* mutation (c.1274A>G|p.Asn425Ser), a *CARD11* mutation (c.2735G>A|p.Arg912Gln), and an *SH2B3* mutation (c.1307A>G|p.Asp436Gly). The *TCF3-ZNF384* fusion was further confirmed by qPCR and fluorescence *in situ* hybridization (FISH) using two break-apart probes for *TCF3* and *ZNF384* (**Figures 1C, D**) (Wuhan Kanglu Biotechnology Co., Ltd).

The patient received chemotherapy according to the JALSG-ALL202-O scheme (8). He achieved complete remission (CR) after induction chemotherapy but relapsed after one cycle of consolidation chemotherapy. Immunophenotyping revealed 29.9% lymphoblasts (mainly positive for CD34, CD19, CD10, CD58, and CD81dim; negative for CD20, CD38, CD123). Relapse leukemia cells from the bone marrow were cultured for 72 hours ex vivo, and a panel of 156 kinds of cytotoxic drugs, targeted therapy drugs, combination chemotherapy drugs, etc. (listed in **Supplementary Table 2**), was used for sensitivity screening. This service was provided by Hefei PreceDo Pharmaceuticals Co., Ltd. Relative inhibition rates higher than 80% were listed in **Figure 1E**. Interestingly, the lymphoblasts showed moderate sensitivity to both ALL schemes (VDLP, CAM, hyper-CVAD (B), etc.) and AML schemes (HAD, DA, MA, etc.) (**Figure 1F**), as well as to multiple targeted therapeutic drugs, such as the Jak2 inhibitor ruxolitinib, the tyrosine kinase inhibitors sorafenib, midostaurin, and imatinib, the proteasome inhibitors carfilzomib (**Figure 1G**). Furthermore, the hyper-CVAD (B) scheme showed multiple synergistic effects with multiple targeted compounds (bortezomib, decitabine, sorafenib and chidamide) (**Figure 1H**) and had the highest inhibition rates (**Figure 1E**). The patient chose the hyper-CVAD (B) scheme combined with sorafenib and gained CR with 0.15% minimal residual disease by flow cytometry (positive for CD19,CD34,CD10,CD123,CD81; negative for CD20,CD38) and 6.11% of *TCF3-ZNF384* by Q-PCR after one cycle of treatment. Then the patient received a second cycle of consolidation chemotherapy with HAD scheme directing against the myeloid lineage (9, 10) and gained molecular CR with negative *TCF3-ZNF384* by qPCR. Currently, the patient is waiting for stem cell transplantation.

## Discussion

*TCF3*, also called *E2A*, can encode several transcription factors *via* alternative splicing and plays important roles in lymphopoiesis in B- and T-cell lineage (11). *ZNF384*, also called *CIZ* or *NMP4*, is a nucleocytoplasmic shuttling protein that associates with focal adhesions and regulates matrix metalloproteinase (MMP) expression (12). The *TCF3-ZNF384* fusion can transform 3T3 cells, and it is the pathogenic basis of leukemia (13). As shown in **Supplementary Table 3**, most *TCF3-ZNF384* fusions contain exon 11 or 13 of *TCF3* and exon 2 or 3 of *ZNF384*, and such fusions retain the transactivation domain of *TCF3* and the entire coding region of *ZNF384*. As such, both loss of function of *TCF3* and transactivation of *ZNF384* target genes may contribute to leukemogenesis; however, transactivation of *ZNF384* is likely more important, as the gene expression profiles of ALL cases with *TCF3-ZNF384* fusion are similar to those with other *ZNF384* rearrangements but different from those with the *TCF3-PBX1* fusion (5). The *TCF3-ZNF384* fusion was first reported by Hunger, SP and Zhong, CH in 2002 in an abstract form (14). Later, it was reported to be present in multiple pediatric B-ALL and B/M MPAL cases. We reviewed the literature and found 43 cases of BCP-ALL (including this case) and 9 cases of MPAL with detailed clinical data, as shown in **Supplementary Table 3** (4–6, 15–25). Only one adult B-ALL case has been reported. The case included a 21-year-old male who relapsed and died 15 months after diagnosis (23). Here, we report a second adult B-ALL patient with the *TCF3-ZNF384* fusion. This patient also showed aberrant expression of myeloid marker CD13 and CD33. However CD10 was expressed in this patient, which accounted only a small part (8/29, **Table 1**) of patients.

**Table 1:** Clinical characteristics of *TCF3-ZNF384* positive ALL and MPAL.

Case characteristics	ALL (N=43)	MPAL (N=9)	*P* value
Gender (M/F)	24/19	6/3	0.717
Age	3 (0.7-41)	6 (0-17)	0.924
WBC (×10^9/L) Median (Range)	25.2 (0.04-150.2)	53 (11.2-1500)	0.161
NCI Risk stratification (HR/SR)	15/19	NA	NA
Complex karyotype/others	5/40	1/6	1.0
5y-OS	0.757±0.088	0.61±0.181	0.552
5y-RFS	0.616±0.105	NA	NA
Lineage switch (positive/all)	3/32	NA	NA
Immunophenotype			
CD10 (negative/all)	21/29	NA	NA
CD33 (positive/all)	25/31	NA	NA
CD13 (positive/all)	11/26	NA	NA

ALL, acute lymphoblastic leukemia; MPAL, mixed phenotype acute leukemia; NCI, National Cancer Institute; HR, high risk; SR, standard risk; OS, overall survival; RFS, relapse free survival; NA, not available or not applicable.

Our case also showed early relapse, which implies poor prognosis. Interestingly, *in vitro* drug sensitivity screening using the relapsed leukemia cells showed sensitivity to both ALL- and AML-directed schemes. By reviewing the literature, we found that the genomic landscapes of B-ALL and MPAL with *ZNF384* rearrangement were similar, and the gene expression profiles of *ZNF384*-rearranged B-ALL and MPAL cases were essentially indistinguishable (6). Under selective pressure, 10.7% (3/28) of *TCF3-ZNF384* B-ALL patients showed lineage switching (**Supplementary Table 3**). Even without selective pressure, transplantation of sorted subpopulations of cells from a *ZNF384*-rearranged cell line showed propagation of immunophenotypic diversity (6). Moreover, the gene expression profile in *TCF3-ZNF384*-positive patients showed enrichment of hematopoietic stem cell features (5), and a case of twins with *ZNF384*-rearranged ALL indicated that fetal hematopoietic progenitor cells are the cell of origin of this disease (26). All of the above findings imply that leukemias with *TCF3-ZNF384* fusion may be derived from hematopoietic stem and progenitor cells, and regardless of immunophenotype, they may have a similar pathogenic basis and clinical characteristics. To further support this hypothesis, we compared the clinical characteristics of *TCF3-ZNF384*-positive ALL and MPAL, as shown in **Table 1**. No statistically significant differences in clinical characteristics such as gender, age, WBC, karyotype and overall survival (OS) were found between the two groups. We further subclassified *TCF3-ZNF384*-rearranged ALL according to the expression of CD10, CD13 and CD33 and compared relapse-free survival (RFS) and OS. Still, we did not find any effects of the immunophenotype on prognosis (all *P >*0.05, data not shown). Even though more cases need to be accumulated, all above suggests that *TCF3-ZNF384*-positive leukemia may be a distinct subtype of leukemia regardless of immunophenotype. Our *in vitro* drug sensitivity screening further supported the above speculation and provided first-hand evidence that *TCF3-ZNF384*-rearranged ALL can be treated with both ALL- and AML-directed schemes. Considering the frequent lineage switches, a uniform strategy directed at both lymphoid and myeloid lineages or at hematopoietic stem cells may be better for *TCF3-ZNF384*-positive leukemia. This patient received lymphoid lineage directed scheme firstly and reached CR, then consolidated with myeloid lineage directed scheme and gained molecular CR, which provided a good model for the treatment of this disease. This idea was further supported by the fact that the two *TCF3-ZNF384* rearranged MPAL patients who received therapy targeting both myeloid and lymphoid lineages had the longest OS among all 9 patients (6).

Notably, our results show moderate sensitivity of leukemia cells to multiple targeted therapeutic drugs, Jak2 inhibitor ruxolitinib, tyrosine kinases inhibitor midostaurin, sorafenib, imatinib and proteasome inhibitor carfilzomib. This phenomenon may be partly related to the multiple concurrent molecular mutations. The patient in this case showed a *FLT3* mutation, which confers sensitivity to midostaurin and sorafenib. *SH2B3* mutation is involved in abnormal activation of the Jak-stat signaling pathway, which provides a molecular mechanism explaining the sensitivity to *Jak2* inhibitors in this patient (27). It in unknown whether leukemias with *ZNF384* rearrangement without these mutations also show sensitivity to these small molecule compounds. In fact, *ZNF384-*rearranged leukemia shows overexpression of *FLT3* and remarkable responsiveness to *FLT3* kinase inhibitors even without *FLT3* mutation (28). JAK/STAT-class and RAS/RAF/MAPK-class aberrations were found in 21% and 43% of *ZNF384* rearrangement patients, respectively (29). Therefore, small molecule targeted therapies may be promising treatment options and deserve further investigation. This patient received tyrosine kinases inhibitor sorafenib combined with hyper-CVAD (B) scheme and achieved CR after only one cycle of treatment. This case provided a good model for individualized accurate treatment under the direction of drug sensitivity screening. Even though the ex-vivo approach may not always work as expected as the pharmacodynamics of each patient contribute to the drug sensitivity and toxicity, it did help guide the treatment of many disease, such as chronic myeloid leukemia (CML) (30), acute myeloid leukemia (AML) (31, 32) and ALL (33).

In conclusion, this study reports a rare case of adult B-ALL with *TCF3-ZNF3* fusion that was characterized by early recurrence. The relapsed leukemia cells were sensitive to both ALL and AML schemes *in vitro*, which supports the viewpoint that *TCF3-ZNF384*-positive leukemia may be a distinct subtype of leukemia regardless of immunophenotype and should be treated with a uniform strategy directed at both lymphoid and myeloid lineages or at hematopoietic stem cells. Small molecule targeted therapies may be promising treatment options and deserve further investigation.

## Data Availability Statement

The original contributions presented in the study are included in the article/**Supplementary Materials**. Further inquiries can be directed to the corresponding author.

## Ethics Statement

Ethical review and approval were not required for the study on human participants in accordance with the local legislation and institutional requirements. The patients/participants provided their written informed consent to participate in this study.

## Author Contributions

XY and DC guided the treatment of this case. NL and LW drafted the manuscript and reviewed all related literature. XY and LW interpreted data and critically revised the manuscript. All authors contributed to the article and approved the submitted version.

## Funding

This study was funded by the National Youth Top-notch Talent of Ten Thousand Talent Program (2014–253) and Translational Research Grant of HCRCH (2020ZKMB06).

## Conflict of Interest

The authors declare that the research was conducted in the absence of any commercial or financial relationships that could be construed as a potential conflict of interest.

## Publisher’s Note

All claims expressed in this article are solely those of the authors and do not necessarily represent those of their affiliated organizations, or those of the publisher, the editors and the reviewers. Any product that may be evaluated in this article, or claim that may be made by its manufacturer, is not guaranteed or endorsed by the publisher.
