# Supplementary material for: Leukemia With TCF3-ZNF384 Rearrangement as a Distinct Subtype of Disease With Distinct Treatments: Perspectives From A Case Report and Literature Review
Source: Front Oncol. 2021 Jul 28;11:709036. doi: 10.3389/fonc.2021.709036 (PMC8357369; doi:10.3389/fonc.2021.709036)
Supplement: Supplementary file 1 [file Table_1.docx]

Supplementary table 1: RT-PCR assay covering 56 commonly detected fusion genes in leukemia

| *AML1-ETO* | *PML-RaRa(V)* | *FIP1L1-RARα* | *AML1-MDS1/EVI1* | *STRNE6-PDGFRA* | *KMT2A-AF1q* |
| --- | --- | --- | --- | --- | --- |
| *BCR-ABL（210）* | *NPM-RARα* | *TEL-AML1* | *AML1-MTG16* | *BCR-PDGFRA* | *KMT2A-AF4* |
| *BCR-ABL（190）* | *NUMA1-RARα* | *E2A-PBX1* | *ETV6-PDGFRA* | *TEL-PDGFRB* | *KMT2A-AF6* |
| *BCR-ABL（230）* | *PLZF-RARα* | *E2A-HLF* | *FIP1L1-PDGFRA* | *KMT2A-AF10* | *KMT2A-AF9* |
| *PML-RaRa（L）* | *PRKAR1A-RARα* | *SIL-TAL1* | *KIF5BE-PDGFRA* | *KMT2A-AF17* | *KMT2A-AFX* |
| *PML-RaRa(S)* | *STAT5b-RARα* | *DEK-CAN* | *CDK5RAP2-PDGFRA* | *KMT2A-AF1p* | *KMT2A-ELL* |
| *NUP98-HoxA13* | *NUP98-HoxA9* | *NUP98-HoxC11* | *NUP98-HoxD13* | *NUP98-PMX1* | *KMT2A-ENL* |
| *TEL-ABL* | *TEL-JAK2* | *TLS-ERG* | *NUP98-HoxA11* | *SET-CAN* | *KMT2A-SEPT6* |
| *WT1* | *HOX11* | *HOX11L2* | *CALM-AF10* | *HLXB9-ETV6* | *KMT2A-AF5* |
| *NPM-ALK* | *NPM-MLF1* |  |  |  |  |

Supplementary table 2: In vitro drug sensitivity screening of relapsed leukemia cells with TCF3-ZNF384 fusion.

| **Drugs** | **Classification** | **Dosage** | **Inhibition rate (%)** |
| --- | --- | --- | --- |
| Dasatinib | molecular targeted therapeutic drugs | 70 mg | 29.07 |
|  |  | 100 mg | 47.22 |
| Nilotinib | molecular targeted therapeutic drugs | 400 mg | 26.29 |
| Imatinib | molecular targeted therapeutic drugs | 400 mg | 56.20 |
|  |  | 600 mg | 61.15 |
| Ibrutinib | molecular targeted therapeutic drugs | 420 mg | 57.75 |
|  |  | 560 mg | 55.21 |
| Ruxolitinib | molecular targeted therapeutic drugs | 15 mg | 43.54 |
|  |  | 20 mg | 59.77 |
|  |  | 25 mg | 62.14 |
| Ixazomib | molecular targeted therapeutic drugs | 4 mg | 56.84 |
| Bortezomib | molecular targeted therapeutic drugs | 1.3 mg/m2 | 51.41 |
| Bosutinib | molecular targeted therapeutic drugs | 500 mg | <1 |
| Carfilzomib | molecular targeted therapeutic drugs | 20 mg/m2 | 52.50 |
|  |  | 27 mg/m2 | 63.41 |
| Midostaurin | molecular targeted therapeutic drugs | 50 mg | 54.51 |
|  |  | 100 mg | 61.23 |
| Ponatinib | molecular targeted therapeutic drugs | 45 mg | 20.33 |
| Venetoclax | molecular targeted therapeutic drugs | 20 mg | 36.12 |
|  |  | 100 mg | 38.79 |
|  |  | 400 mg | 43.33 |
| Sorafenib | molecular targeted therapeutic drugs | 400 mg | 62.67 |
| Gilteritinib （ASP2215） | molecular targeted therapeutic drugs | 120 mg | 19.78 |
| Quizartinib | molecular targeted therapeutic drugs | 30 mg | 27.83 |
|  |  | 60 mg | 33.21 |
| Chidamide | molecular targeted therapeutic drugs | 30mg | 49.60 |
| Busulfan | chemotherapy regimens | 0.8 mg/kg | <1 |
| Chloroambucil | chemotherapy regimens | 0.1 mg/kg | <1 |
|  |  | 0.2 mg/kg | 12.01 |
| Melphalan | chemotherapy regimens | 10 mg/m2 | 15.07 |
| Procarbazine | chemotherapy regimens | 50 mg | 23.35 |
| Cyclophosphamide | chemotherapy regimens | 500 mg/m2 | 40.90 |
|  |  | 1000 mg/m2 | 45.49 |
| Isophosphamide | chemotherapy regimens | 1200 mg/m2 | 27.80 |
|  |  | 2500 mg/m2 | 42.67 |
| Bendamustine | chemotherapy regimens | 100 mg/m2 | 45.73 |
|  |  | 120 mg/m2 | 53.78 |
| Lomustine | chemotherapy regimens | 100 mg/m2 | 3.20 |
|  |  | 130 mg/m2 | 8.01 |
| Carmustine | chemotherapy regimens | 100 mg/m2 | 29.24 |
| Dacarbazine | chemotherapy regimens | 200 mg/m2 | 42.05 |
|  |  | 400 mg/m2 | 45.59 |
| Methotrexate | chemotherapy regimens | 10 mg | <1 |
| Cytarabine | chemotherapy regimens | 100 mg/m2 | 41.70 |
|  |  | 2000 mg/m2 | 52.43 |
|  |  | 3000 mg/m2 | 69.15 |
| Fludarabine | chemotherapy regimens | 25 mg/m2 | 66.60 |
| Gemcitabine | chemotherapy regimens | 1000 mg/m2 | 53.82 |
|  |  | 1200 mg/m2 | 67.64 |
| Cladribine | chemotherapy regimens | 0.09 mg/kg | 44.27 |
| Clofarabine | chemotherapy regimens | 50 mg/m2 | 61.07 |
| Hydroxyurea | chemotherapy regimens | 20 mg/kg | 4.98 |
|  |  | 60 mg/kg | <1 |
| Mercaptopurine | chemotherapy regimens | 80 mg/m2 | 4.90 |
|  |  | 100 mg/m2 | <1 |
| 5-Fluorouracil | chemotherapy regimens | 250 mg | <1 |
|  |  | 750 mg | <1 |
| Decitabine | chemotherapy regimens | 10 mg/m2 | <1 |
|  |  | 15 mg/m2 | <1 |
|  |  | 20 mg/m2 | 12.70 |
| Azacitidine | chemotherapy regimens | 75 mg/m2 | 56.81 |
|  |  | 100 mg/m2 | 67.61 |
| Docetaxel | chemotherapy regimens | 75 mg/m2 | 4.79 |
| Vindesine | chemotherapy regimens | 3 mg/m2 | 23.68 |
| Vincristine | chemotherapy regimens | 1.4 mg/m2 | 20.20 |
| Vinblastine | chemotherapy regimens | 10 mg | 28.50 |
| Vinorelbine | chemotherapy regimens | 30 mg/m2 | 62.02 |
| Paclitaxel | chemotherapy regimens | 135 mg/m2 | 61.10 |
|  |  | 200 mg/m2 | 61.83 |
| Hydroxycamptothecin | chemotherapy regimens | 8 mg | 61.69 |
| Teniposide | chemotherapy regimens | 30 mg/m2 | 13.23 |
|  |  | 60 mg/m2 | 17.43 |
| Topotecan | chemotherapy regimens | 1.25 mg/m2 | 52.87 |
| Etoposide | chemotherapy regimens | 60 mg/m2 | 57.35 |
|  |  | 100 mg/m2 | 60.01 |
| Irinotecan | chemotherapy regimens | 350 mg/m2 | <1 |
| Idarubicin | chemotherapy regimens | 7 mg/m2 | 51.59 |
|  |  | 8 mg/m2 | 61.94 |
| Daunorubicin | chemotherapy regimens | 40 mg/m2 | 48.59 |
|  |  | 60 mg/m2 | 50.91 |
|  |  | 90 mg/m2 | 62.38 |
| Aclacinomycin | chemotherapy regimens | 0.4 mg/kg | 5.68 |
|  |  | 1 mg/kg | 39.34 |
| Pirarubicin | chemotherapy regimens | 20 mg/m2 | 55.02 |
|  |  | 40 mg/m2 | 67.16 |
| Epirubicin | chemotherapy regimens | 60 mg/m2 | 55.15 |
|  |  | 120 mg/m2 | 67.67 |
| Doxorubicin | chemotherapy regimens | 40 mg/m2 | 47.29 |
|  |  | 60 mg/m2 | 50.87 |
| Mitoxantrone | chemotherapy regimens | 8 mg/m2 | 53.59 |
|  |  | 14 mg/m2 | 60.73 |
| Mitomycin | chemotherapy regimens | 10 mg | 59.54 |
|  |  | 20 mg | 66.86 |
| Bleomycin | chemotherapy regimens | 15 mg | 57.91 |
|  |  | 30 mg | 68.90 |
| Oxaliplatin | chemotherapy regimens | 130 mg/m2 | 38.80 |
| Carboplatin | chemotherapy regimens | 50 mg/m2 | 11.53 |
|  |  | 400 mg/m2 | 51.42 |
| Cisplatin | chemotherapy regimens | 80 mg/m2 | 64.28 |
|  |  | 120 mg/m2 | 60.95 |
| Arsenic trioxide | chemotherapy regimens | 7 mg/m2 | 35.07 |
| Amsacrine | chemotherapy regimens | 120 mg/m2 | 52.33 |
| Desferrioxamine B | chemotherapy regimens | 20 mg/kg | 17.10 |
| Deferasirox | chemotherapy regimens | 20 mg/kg | 48.31 |
| Tretinoin | chemotherapy regimens | 10 mg | 16.97 |
| Bexarotene | chemotherapy regimens | 300 mg/m2 | 15.43 |
|  |  | 400 mg/m2 | 11.46 |
| Homoharringtonie | chemotherapy regimens | 1 mg | 58.99 |
|  |  | 4 mg | 63.99 |
| Adefovir Dipivoxil | chemotherapy regimens | 10 mg | 20.75 |
| Dexamethasone | glucocorticoid | 40 mg/m2 | 42.54 |
| Methylprednisolone | glucocorticoid | 40 mg | 44.33 |
| Prednisone | glucocorticoid | 60 mg | <1 |
|  |  | 80 mg | <1 |
| Hydrocortisone | glucocorticoid | 100 mg | 16.92 |
| Pomalidomide | immunoregulant | 4 mg | 29.30 |
| Lenalidomide | immunoregulant | 10 mg | 18.81 |
|  |  | 25 mg | 6.53 |
| Thalidomide | immunoregulant | 25 mg | 10.23 |
|  |  | 50 mg | <1 |
|  |  | 100 mg | <1 |
| Mycophenolate Mofetil | immunoregulant | 0.5 g | 7.40 |
|  |  | 1.5 g | 16.39 |
| ABVD | chemotherapy regimens | Doxorubicin 25 mg/m2  Bleomycin 10 mg/m2  Vincristine 6 mg/m2  dacarbazine 375 mg/m2 | 11.69 |
| CAG | chemotherapy regimens | cytarabine 10 mg/m2  aclacinomycin 20 mg | 59.89 |
| CAM | chemotherapy regimens | Cyclophosphamide 750 mg/m2  cytarabine 100 mg/m2  6-mercaptopurine 60 mg/m2 | 67.69 |
| CHOEP | chemotherapy regimens | Cyclophosphamide 750 mg/m2  Epirubicin 70 mg/m2  Vincristine 1.4 mg/m2   etoposide 100 mg/m2  prednison; 60 mg/m2 | 62.37 |
| CHOP | chemotherapy regimens | Cyclophosphamide 750 mg/m2  Doxorubicin 50 mg/m2  Vincristine 1.4 mg/m2  prednison; 60 mg/m2 | 36.13 |
| CLAG | chemotherapy regimens | Cladribine 5 mg/m2  cytarabine 2000 mg/m2 | 57.23 |
| COATD | chemotherapy regimens | Cyclophosphamide 750 mg/m2 Vincristine 1.4 mg/m2  cytarabine 100 mg/m2 teniposide 100 mg/m2  dexamethasone 6 mg/m2 | 49.39 |
| COP | chemotherapy regimens | Vincristine 1.4 mg/m2  Cyclophosphamide 750 mg/m2  prednison; 60 mg/m2 | 20.04 |
| DA | chemotherapy regimens | Daunorubicin 60 mg/m2  cytarabine 100 mg/m2 | 74.65 |
| 6-MP+MTX | chemotherapy regimens | 6-mercaptopurine 60 mg/m2  Methotrexate 20 mg/m2 | 13.00 |
| DAC | chemotherapy regimens | Cladribine 5 mg/m2  cytarabine 200 mg/m2  Daunorubicin 60 mg/m2 | 72.61 |
| DA-EPOCH | chemotherapy regimens | etoposide 50 mg/m2  Vincristine 0.4 mg/m2 Doxorubicin 10 mg/m2  Cyclophosphamide 750 mg/m2 | 55.95 |
| DCAG | chemotherapy regimens | Decitabine 15 mg/m2  cytarabine 10 mg/m2  aclacinomycin 8 mg/m2 | 65.23 |
| DHAP | chemotherapy regimens | dexamethasone 40 mg  cytarabine 2000 mg/m2  cisplatin 100 mg/m2 | 75.84 |
| DOLP | chemotherapy regimens | Vincristine 2 mg  Daunorubicin 60 mg/m2  prednisone 60 mg  L-ASP 10000 u | 63.89 |
| ESHAP | chemotherapy regimens | etoposide 60 mg/m2  Methylprednisolone; 500 mg/m2  cytarabine 2 g/m2  cisplatin 25 mg/m2 | 74.91 |
| FLAG | chemotherapy regimens | Fludarabine 30 mg/m2  cytarabine 2 g/m2 | 75.34 |
| GDP | chemotherapy regimens | gemcitabine 1 g/m2  dexamethasone 40 mg  cisplatin 75 mg/m2 | 60.95 |
| HA | chemotherapy regimens | Homoharringtonine 2.5 mg/m2  cytarabine 100 mg/m2 | 77.18 |
| HAA | chemotherapy regimens | Homoharringtonine 2 mg/m2  cytarabine 100 mg/m2  aclacinomycin 20 mg | 83.30 |
| HAD | chemotherapy regimens | Homoharringtonine 2 mg/m2  cytarabine 100 mg/m2 Daunorubicin 40 mg/m2 | 83.08 |
| HD-DA | chemotherapy regimens | cytarabine 3000 mg/m2  Daunorubicin 30 mg/m2 | 75.16 |
| Hyper-CVAD(A) | chemotherapy regimens | Cyclophosphamide 300 mg/m2  Doxorubicin 50 mg/m2 Vincristine 1.4 mg/m2  dexamethasone 40 mg/d | 43.79 |
| Hyper-CVAD(B) | chemotherapy regimens | methotrexate 800 mg/m2  cytarabine 3000 mg/m2 | 59.39 |
| IOLP | chemotherapy regimens | Vincristine 1.4 mg/m2  idarubicin 10 mg  prednisone 60 mg/m2  L-ASP 10000 u | 31.43 |
| IA | chemotherapy regimens | idarubicin 12 mg/m2 cytarabine 100 mg/m2 | 67.43 |
| ICE | chemotherapy regimens | etoposide 100 mg/m2   Carboplatin 800 mg  Ifosfamide 5 g/m2 | 73.80 |
| MA | chemotherapy regimens | Mitoxantrone 12 mg/m2  cytarabine 200 mg/m2 | 74.10 |
| MINE | chemotherapy regimens | Ifosfamide 1333 mg/m2  Mitoxantrone 8 mg/m2  etoposide 65 mg/m2 | 62.19 |
| MOACD | chemotherapy regimens | Mitoxantrone 8 mg/m2  Vincristine 1.4 mg/m2  Cyclophosphamide 600 mg/m2  cytarabine 100 mg/m2  dexamethasone 6 mg/m2 | 68.17 |
| VDLD | chemotherapy regimens | Vincristine 1.5 mg/m2  Daunorubicin 30 mg/m2  dexamethasone 8 mg/m2 L-ASP 5000 u/m2 | 63.23 |
| VDLP | chemotherapy regimens | Vincristine 1.5 mg/m2  Daunorubicin 30 mg/m2  prednison; 40 mg/m2  L-ASP 5000 u/m2 | 60.72 |
| VTD | chemotherapy regimens | Bortezomib 1.3 mg/m2  Thalidomide 200 mg/d  dexamethasone 20 mg | 74.52 |
| HOAP | chemotherapy regimens | Homoharringtonine 4 mg/d  Vincristine 2 mg/d  cytarabine100 mg/d  prednisone40 mg/d | 70.63 |
| DAT | chemotherapy regimens | Daunorubicin 25 mg/m2  cytarabine 100 mg/m2  6-mercaptopurine 100 mg/m2 | 63.31 |
| ME | chemotherapy regimens | Mitoxantrone 10 mg/m2  etoposide 100 mg/m2 | 54.90 |
| DAE | chemotherapy regimens | Daunorubicin 40 mg/m2  cytarabine 100 mg/m2   etoposide 100 mg/m2 | 52.56 |
| IAE | chemotherapy regimens | idarubicin 12 mg/m2  cytarabine 200 mg/m2   etoposide 100 mg/m2 | 56.52 |
| Mm | chemotherapy regimens | methotrexate 3 g/m2  6-mercaptopurine 25 mg/m2 | 43.40 |
| MOAP | chemotherapy regimens | cytarabine 1.0 g/m2  Mitoxantrone 10 mg/d  Vincristine 2 mg/d  prednisone 60 mg/d | 67.92 |
| DOMP | chemotherapy regimens | Vincristine 2 mg/d  Daunorubicin 60 mg  6-mercaptopurine 50 mg/d  prednisone 2 mg/kg/d | 66.21 |
| FA+VP | chemotherapy regimens | Fludarabine 30 mg/m2  cytarabine 2 g/m2  Vincristine 2 mg | 68.85 |
| IOAP | chemotherapy regimens | Vincristine 1.4 mg/m2  idarubicin 10 mg/d  prednisone 60 mg/m2  cytarabine 100 mg/m2 | 75.12 |
| TA | chemotherapy regimens | teniposide 100 mg/m2  cytarabine 100 mg/m2 | 65.87 |
| Large dose MTX+L-ASP | chemotherapy regimens | methotrexate 3 g/m2  L-asparaginas 6000 IU/m2 | 29.67 |
| VDCLP | chemotherapy regimens | Vincristine 1.4 mg/m2  Daunorubicin 40 mg/m2  Cyclophosphamide 750 mg/m2  L-asparaginas 6000 IU/m2  prednison; 1 mg/kg/d | 42.51 |
| AZA+HAG | chemotherapy regimens | Azacitidine 75 mg/m2  Homoharringtonine 1 mg/d  cytarabine 10 mg/m2 | 51.28 |
| DEC+DAG | chemotherapy regimens | Decitabine 20 mg/m2  Daunorubicin 40 mg/m2  cytarabine 30 mg/m2 | 46.50 |
| DEC+IA | chemotherapy regimens | Decitabine 20 mg/m2  cytarabine 25 mg  idarubicin 10 mg | 56.95 |
| AZA+DAG | chemotherapy regimens | Azacitidine 75 mg/m2 Daunorubicin 20 mg/m2 cytarabine 15 mg/m2 | 61.36 |
| ruxolitinib+Mm | chemotherapy regimens | ruxolitinib 25 mg methotrexate 3 g/m2  6-mercaptopurine 25 mg/m2 | 40.73 |
| Venetoclax+Mm | chemotherapy regimens | Venetoclax400 mg methotrexate 3 g/m2  6-mercaptopurine 25 mg/m2 | 50.36 |
| chidamide+Mm | chemotherapy regimens | chidamide 30 mg methotrexate 3 g/m2  6-mercaptopurine 25 mg/m2 | 19.27 |
| DEC+Mm | chemotherapy regimens | Decitabine 20 mg/m2  methotrexate 3 g/m2  6-mercaptopurine 25 mg/m2 | 46.61 |
| Bortezomib+Mm | chemotherapy regimens | Bortezomib 1.3 mg/m2 methotrexate 3 g/m2  6-mercaptopurine 25 mg/m2 | 83.54 |
| Sorafenib+Mm | chemotherapy regimens | Sorafenib 400 mg methotrexate 3 g/m2  6-mercaptopurine 25 mg/m2 | 76.17 |
| Midostaurin+Mm | chemotherapy regimens | Midostaurin 100 mg methotrexate 3 g/m2  6-mercaptopurine 25 mg/m2 | 79.96 |
| ruxolitinib+FA+VP | chemotherapy regimens | ruxolitinib 25 mg Fludarabine 30 mg/m2  cytarabine 2 g/m2  Vincristine 2 mg | 62.78 |
| Venetoclax+FA+VP | chemotherapy regimens | Venetoclax 400 mg Fludarabine 30 mg/m2  cytarabine 2 g/m2  Vincristine 2 mg | 65.59 |
| chidamide+FA+VP | chemotherapy regimens | chidamide 30 mg Fludarabine 30 mg/m2  cytarabine 2 g/m2  Vincristine 2 mg | 57.85 |
| Decitabine+FA+VP | chemotherapy regimens | Decitabine 20 mg/m2 Fludarabine 30 mg/m2  cytarabine 2 g/m2  Vincristine 2 mg | 58.72 |
| Bortezomib+FA+VP | chemotherapy regimens | Bortezomib 1.3 mg/m2 Fludarabine 30 mg/m2  cytarabine 2 g/m2  Vincristine 2 mg | 53.91 |
| Sorafenib+FA+VP | chemotherapy regimens | Sorafenib400 mg Fludarabine 30 mg/m2  cytarabine 2 g/m2  Vincristine 2 mg | 56.77 |
| Midostaurin+FA+VP | chemotherapy regimens | Midostaurin 100 mg Fludarabine 30 mg/m2  cytarabine 2 g/m2  Vincristine 2 mg | 50.91 |
| ruxolitinib+hyperCVAD(B) | chemotherapy regimens | ruxolitinib 25 mg methotrexate 800 mg/m2  cytarabine 3000 mg/m2 | 71.82 |
| Venetoclax+hyperCVAD(B) | chemotherapy regimens | Venetoclax 400 mg methotrexate 800 mg/m2  cytarabine 3000 mg/m2 | 80.32 |
| chidamide+hyperCVAD(B) | chemotherapy regimens | chidamide 30 mg methotrexate 800 mg/m2  cytarabine 3000 mg/m2 | 84.14 |
| Decitabine+hyperCVAD(B) | chemotherapy regimens | Decitabine 20 mg/m2 methotrexate 800 mg/m2  cytarabine 3000 mg/m2 | 85.80 |
| Bortezomib+hyperCVAD(B) | chemotherapy regimens | Bortezomib 1.3 mg/m2 methotrexate 800 mg/m2  cytarabine 3000 mg/m2 | 86.86 |
| Sorafenib+hyperCVAD(B) | chemotherapy regimens | Sorafenib 400 mg methotrexate 800 mg/m2  cytarabine 3000 mg/m2 | 85.70 |
| Midostaurin+hyperCVAD(B) | chemotherapy regimens | Midostaurin 100 mg methotrexate 800 mg/m2  cytarabine 3000 mg/m2 | 77.34 |
| Sorafenib+ruxolitinib | chemotherapy regimens | Sorafenib 400 mg ruxolitinib 25 mg | 54.55 |
| Midostaurin+ruxolitinib | chemotherapy regimens | Midostaurin 100 mg ruxolitinib 25 mg | 58.34 |
| Sorafenib+Venetoclax | chemotherapy regimens | Sorafenib 400 mg Venetoclax 400 mg | 57.64 |
| Midostaurin+Venetoclax | chemotherapy regimens | Midostaurin 100 mg Venetoclax 400 mg | 58.37 |
| Decitabine+chidamide | chemotherapy regimens | Decitabine 20 mg/m2 chidamide 30 mg | 55.74 |
| Sorafenib+ruxolitinib+Bortezomib | chemotherapy regimens | Sorafenib 400 mg ruxolitinib 25 mg Bortezomib 1.3 mg/m2 | 61.44 |
| Sorafenib+ruxolitinib+Venetoclax | chemotherapy regimens | Sorafenib 400 mg ruxolitinib 25 mg Venetoclax 400 mg | 68.38 |
| Sorafenib+ruxolitinib+chidamide | chemotherapy regimens | Sorafenib 400 mg ruxolitinib 25 mg chidamide 30 mg | 47.86 |
| Sorafenib+ruxolitinib+Decitabine | chemotherapy regimens | Sorafenib 400 mg ruxolitinib 25 mg Decitabine 20 mg/m2 | 57.16 |

Supplementary table 3. Clinical characteristics of 43 cases of ALL and 9 cases of MPAL with TCF3-ZNF384 fusion.

|  | Case No. | Frequency | Country | S/A | WBC(*10^9/L) | NCI Risk | CNS infiltration | Lineage Switch | Immunophenotype（EGIL） | Karyotype | Fusion position | Gene mutation or other fusion | Prognosis |
| --- | --- | --- | --- | --- | --- | --- | --- | --- | --- | --- | --- | --- | --- |
| Kapadia, A. B.（16） | 1 | NA | India | F/2 | 14.7 | SR | NA | ALL-MPAL, B/myeloid | CD19bri, cCD79a, cCD22 and CD33,CD10dim,CD13dim | NA | NA | NA | NA |
| Ross A. Rowsey（17） | 2 | NA | USA | F/2 | NA | NA | NA | NA | NA | 46,XX,t(16;16)(p13.3;q22),add(19)(p13.2)[16]/46,XX[4] | e13;e3 | NA | NA |
|  | 3 |  |  | M/6 | NA | NA | NA | NA | NA | NA | e17;e7 | NA | NA |
|  | 4 |  |  | F/8 | NA | NA | NA | NA | NA | NA | e13;e3 | NA | NA |
|  | 5 |  |  | F/13 | NA | NA | NA | NA | NA | 48,X,add(X)(q13),+2,der(5)add(5)(p12)t(5;10)(q31;q22),+8,idic(9) (p10),der(10)t(5;10)(q31;q22),t(12;14)(q11;p11.2),add(15)(q22), add(19)(p13.3),+mar[18]/46,XX[2] | e13;e3 | NA | NA |
|  | 6 |  |  | F/1 | NA | NA | NA | NA | NA | 46,XX,add(19)(p13.3)[8]/46,idem,add(5)(q13),del(14)(q13)[1]/46, XX[11] | e11;e3 | NA | NA |
|  | 7 |  |  | M/8 | NA | NA | NA | NA | NA | 46,XY,der(3)(3pter->3q13.2::3q26.3->3qter),der(9)(9pter>9p22::3q13.2->3q26.2::9p13->9qter)[5]/46,XY[15] | e13;e2 | NA | NA |
| Nishimura, A.（18） | 8 | NA | Japan | F/4 | NA | NA | NA | No | CD19+,CD34+,CD33+,CD10dim+,cMPO-,CD14- | 46,XX | NA(at relapse) |  | Relapse at 10 years later,underwent unrelated cord blood transplantation, CR 14 months after transplantation |
|  | 9 |  |  | F/18 | NA | SR | NA | No | at relapse: CD33+, CD38+, HLA-DR+, CD10- | 46, XX, der(13;14)(q10;q10), +5, add(19)(p13) | NA(at relapse) |  | Relapse 13 years after diagnosis,underwent unrelated bone marrow transplantation with MAC,relapsed 3 years after transplantation and died from a transplant-related complication after a second transplantation. |
| Bueno, C.  （19） | 10 | NA | Spain | M/0.7 | 0.15 | SR | CNS1 | NA | CD45lowCD34++CD19+CD10-CD33low | 46XY,add(12)p(13) |  | 227 mutations including PTPN1, | Alive in CR 18 months |
|  | 11 |  |  | M/0.7 | 0.038 | SR | CNS3 | NA | CD45lowCD34++CD19+CD10-CD33low | 46XY,add(12)p(13) |  | 261 mutations including PTPN1, | Alive 18 in CR months |
| Oberley, M. J.  （26） | 12 | NA | USA | M/1.1 | 0.3 | SR | CNS2 | B-ALL-AML | positive for CD19, CD20 (partial), CD22, CD34, CD38, CD13, CD33 (partial), and HLA-DR; negative for CD10,MPO, and TdT; | at diagnosis: 46,XY,del(12)(p13) [3]/46,XY[27]; 46,XY[1]//46,XX[19] (posttransplant chimerism); Secondry relapse: 46,XY[20] | e11;e2 | FLT3-ITD(when AML relapse) | Relapse 3 months after diagnosis; twice CAR-T, HSCT, CAR-T again, second HSCT,8 months later AML relapse,died 4 months after the last relapse |
| Zaliova, M.  （28） | 13 | NA | Czech Republic | M/3 | 32.3 | SR | NA | No | CD27pos/CD44low-neg | SNP array：Gain (1q21.1qterm),Gain(2q11.1qterm),Loss(4ptermp15.1), Loss(9p24.2),Loss(13q14.2), Gain(22q11.22qterm) | 12p13.31;19p13.3 | SEPSECS,SPEN,NKD2,FN1,HUS1B,NF1, KRT6B,DHCR24,SLC22A9,DPP9-AS1 |  |
| Qian, M.（25） | 14 | 3/231 in paediatric ALL;  3/204 in paediatric B-ALL | Asian | M/2.1 | 140 | HR | NA | NA | HLA-DR+/CD34+/CD10-/CD19+/CD22+/CD79a+ | 46,XY[16] | e17;e7 | DUSP7，PPP1R16A，PTPN11，MYH8 | NA |
|  | 15 |  |  | F/2.3 | 57 | HR | NA | NA | HLA-DR+/CD34+/CD38+/CD10-/CD19+/CD20-/CD22dim/cyCD79a+/cyIgM-/cyCD3-/CD2-/CD5-/CD7-/CD13+/CD15dim/CD33+/MPO- | 46,XX[20] | e11;e2 | MYCN，SLC23A1 | NA |
|  | 16 |  |  | M/5.5 | 5.5 | SR | NA | NA | HLA-DR+/CD34+/CD38+/CD10-/CD19+/CD20-/CD22dim/cyCD79adim/cyIgM-/cyCD3-/CD2-/CD5-/CD7-/CD13dim/CD15dim/CD33+/MPO- | 46,XY,add(16)(q24)[1]/46,XY[19] | e17;e7 | CHD8,BCL11B,CREBBP | NA |
| Marincevic-Zuniga, Y.  （20） | 17 | 2/134 in paediatric ALL;  2/116 in paediatric B-ALL | Sweden | M/10.5 | 20.6 | HR | NA | No | NA | 46,XY,del(7)(q22)[5]/46,XY[15].ish.t(12;19)(?;p13) | NA |  | 5.6y relapse |
|  | 18 |  |  | M/10.9 | 10.9 | HR | NA | No | NA | 46,XY,del(7)(q22)[8]/46,XY,del(6)(q2?1)[7]/46,XY[17] | NA | VASH2-ATF3;CD69-HIST1H2BG | 8y CR1 Alive |
| Hirabayashi, S.  （5） | 19 | 7/291 in paediatric B-ALL;  7/130 in paediatric B-others | Japan | F/2 | 36.6 | SR | CNS1 | No | positive for cyt-CD79a,HLA-DR,CD19,CD22,CD24,CD34,CD13,CD33,negative for CD10 | 46,XX | e11;e3 | NA | 1st CR, Alive 3.5y |
|  | 20 |  |  | M/9 | 3.2 | SR | CNS1 | No | positive for cyt-CD79a,HLA-DR,CD19,CD22,CD24,CD34,cyt-TdT, negative for CD10,CD33,CD13 | 46,XY | e13;e3 | NA | 4.7y Relapse, SCT, Alive 7y |
|  | 21 |  |  | M/3 | 137.26 | HR | CNS1 | No | positive for cyt-CD79a,HLA-DR,CD19,CD22,CD24,CD34,cyt-TdT, CD33, negative for CD10,CD13 | 46,XY | e17;e7 | NA | 1st CR, Alive 6.1y |
|  | 22 |  |  | M/2 | 4 | SR | CNS1 | No | positive for cyt-CD79a,HLA-DR,CD19,CD22,cyt-Igμ,CD24,CD34,cyt-TdT,CD99, negative for CD10,CD13,CD33 | 46,XY | e13;e3 | Neg | 1st CR, Alive 6.4y |
|  | 23 |  |  | F/1 | 21.1 | SR | CNS1 | No | positive for HLA-DR,CD19,CD24,CD10,CD34, negative for cyt-TdT, CD13,CD33 | 46,XX | e13;e3 | MLL2,ASH1L | 1st CR, Alive 4.5y |
|  | 24 |  |  | F/9 | 2.75 | SR | CNS1 | No | positive for HLA-DR,CD19,CD22,CD24,CD34, CD33, negative for CD10,CD13 | NA | e13;e3 | NA | 1st CR, Alive 2.0y |
|  | 25 |  |  | M/1 | 49.97 | SR | CNS1 | No | positive for cyt-CD79a,HLA-DR,CD19,CD22,CD24,CD34,cyt-TdT, CD13,CD33,negative for CD10 | 46,XY | e13;e3 | PTPN11,ADAMTS9,VWDEMED12 | 1.6y Relapse, SCT, Death 2.5y |
|  | 26 |  |  | F/11 | 1.22 | HR | CNS1 | No | positive for cyt-CD79a,HLA-DR,CD19,CD22,CD24,CD10,CD34,cyt-TdT,CD2negative for CD13,CD33 | 46,XX | e17;e7 | NA | 1st CR, Alive 6.7y |
|  | 27 |  |  | F/2 | 150.2 | HR | CNS1 | No | positive for cyt-CD79a,HLA-DR,CD19,CD22,CD24,CD34, CD13,CD33,negative for CD10 | 46,XX | e11;e3 | NA | 1st CR, Alive 3.6y |
|  | 28 |  |  | F/10 | 4.8 | HR | CNS1 | No | positive for cyt-CD79a,HLA-DR,CD19,CD24,CD34,cyt-TdT,CD99,CD33,negative for CD10,CD13 | 46,XX | e16;e2 | NA | 1st CR, Alive 0.7y |
|  | 29 |  |  | F/3 | 130.9 | HR | CNS1 | No | positive for cyt-CD79a,HLA-DR,CD19,CD22,CD24,CD10,CD34,cyt-TdT,CD99,CD33, negative for CD13 | 46,XX | e17;e7 | NA | 3.4y Relapse, SCT, Alive 4.3y |
|  | 30 |  |  | M/2 | 49.16 | SR | CNS1 | No | positive for cyt-CD79a,HLA-DR,CD19,CD22,CD24,CD34,CD99,CD33,CD11b,negative for CD10,cyt-TdT,CD13 | 46,XY | e13;e3 | NA | 2.9y Relapse, SCT, Death 3.6y |
|  | 31 |  |  | M/2 | 27.45 | SR | CNS1 | No | positive for cyt-CD79a,HLA-DR,CD19,CD22,CD24,CD10,cyt-TdT,CD99,CD33, negative for CD34,CD13,CD33 | 46,XY | e11;e3(at relapse) | MYCN,NRAS,OBSCN,FAT4,KIAA1598,EZH2,MLL2,COL14A1 | 1.6y Relapse, SCT, Death 2.4y |
|  | 32 |  |  | M/3 | 105.9 | HR | CNS1 | No | positive for cyt-CD79a,HLA-DR,CD19,CD22,CD24,CD34,CD99,CD33,negative for CD10,cyt-TdT,CD13 | 46,XY | e13;e3 | KRAS,PTPN11,MLL2 | 1st CR, Alive 2.4y |
|  | 33 |  |  | M/8 | 76.5 | HR | CNS2 | No | positive for cyt-CD79a,HLA-DR,CD19,CD22,CD24,CD10,CD34,cyt-TdT,CD99,CD33,negative for CD13, | 46,XY | e13;e2 | ASH1L,TRERF1, KRT18 | 1st CR, Alive 2.3y |
| Shago, M.  （21） | 34 | 4/240 in paediatric B-ALL | Canada | F/4 | 78.3 | HR | CNS2 | No | CD10-,CD13+;CD33+ | 46,XX,der(12)del(12)(p13) t(12;19)(q24.3;p13.1), der(19)t(12;19)(q24.3;p13.3), der(19)t(19;19)(p13.1;p13.3).ish der(12)(3′TCF3+,5′ZNF384+), der(19)(3′ZNF384+,5′TCF3+) | NA |  | CR 9 years |
|  | 35 |  |  | F/4 | 25.2 | SR | CNS1 | No | CD10–, CD33+ | 46,XX.ish t(12;19)(p13;p13.3)(3′TCF3+,5′ZNF384+; 3′ZNF384+,5′TCF3+) | NA |  | CR 9.2 years |
|  | 36 |  |  | F/2 | 15 | SR | CNS1 | No | CD10–, CD13+, CD33+,MPO+ | 46,XX.ish t(12;19)(p13;p13.3)(3′TCF3+, 5′ZNF384+;3′ZNF384+,5′TCF3+) | NA |  | CR 8.7 years |
|  | 37 |  |  | M/4 | 3.6 | SR | CNS1 | No | CD10–, CD13+, CD33+ | 46,XY,del(9)(q21q32).ish t(12;19) (p13;p13.3)(3’TCF3+, 5’ZNF384+;3’ZNF384+, 5’TCF3+) | NA |  | CR 6.3 years |
| Liu, Y. F.（4） | 38 | 2/199 in paediatric B-ALL;  0/177 in adult B-ALL | China | F/2.3 | 104 | HR | NA | NA | CD13-;CD33+ | 46,XX | NA | LPHN2 p.V320A;COL5A2 p.G612R;ZNF384-RNF180 | Relapse |
|  | 39 |  |  | F/2.2 | 35.1 | SR | NA | NA | CD13-;CD33+ | 46,XY | p13.3;p13.31 | NRAS p.Q61H;FRMD4A p.P564T;PLK5-ATN1 | Relapse |
| C-h Zhong（22） | 40 | NA | USA | M/5 | NA | NA | NA | B-ALL-AML | HLA-DR+, CD19+, CD34+,TdT+ | 46,XY,add(1)(q32),t(9;16)(p21;q22),t(12;19)(p13.3;p13.3), t(13;14)(q14;q32) [21]/46,idem,del(4)(q22q2?5)[5]/46,XY,add(1)(q32),der(9)t(9;16)(p21;q22) t(9;22)(q34;q11.2),t(12;19)(p13.3;p13.3),t(13;14) (q14;q32),der(16)t(9;16)(p21;q22),der(22)t(9;22)(q34;q11.2)[1]/46,XY[3](initial diagnosis);46,XY,add(1)(q32),add(7)(q33),t(9;16)(p21;q22), t(12;19)(p13.3;p13.3),t(13;14)(q14;q32)[19](at relapse) | NA | NA | AML relapse at 4 months after diagnosis,HST,Died 32 days after HST |
| Barber, K. E.（23） | 41 | 1/161 in paediatric B-ALL | UK | M/2 | NA | NA | NA | NA | NA | 46,XY,dup(1q) | NA |  |  |
| La Starza, R.（24） | 42 | NA | Italy | M/21 | 2.8 | HR | NA | No | CD19+ | 46,XY,add(10)(p?),t(12;19)(p13;p13),+13[6]/ 46,XY[11] | NA | NA | Relapse and died 15 months after diagnosis |
| Na Lin  （this case) | 43 | NA | China | M/41 | 150 | HR | NA | No | mainly positive for CD34, CD19, CD10, Ccd79a, TdT, CD22, HLA-DR, CD58, CD13, partially positive for CD38, CD123, weak positive for CD33 and negative for CD117, CD7, CD3, MPO, Ccd3, CD56, CD15, CD79a, cIgM, CD25, CD20, Kappa, Lambda, | 46, XY [20] | e13-e3 | FLT3,TCF3,NOTCH2,CARD11,SH2B3, | Relapse 0.22y after diagnosis, Alive 0.36y |
| Alexander, T. B.(6) | 1 | 9/115 in paediatric ALAL | USA | M/1 | 75.1 | HR | Negative | NA | B/M | 46,Y,-X,add(12)(p11.2),19,+mar1,+mar2 [10/20], 46XY [10/20] | e14;e2 | KRAS,RNFT2,SRCAP | Died 2.8y |
|  | 2 |  |  | F/17 | 43.2 | HR | NA | NA | B/M | 46,XX,t(2;14)(p?11.2;q11.2)[13]/46,XX[7] | e11;e2 | ADRB1,CACNA1F,CFTR,DNAH17,FBXL18,KMT2D,MSC,MYCN,NDST2,RNF11,SMARCA4,ZNF608 | Alive 8.7y |
|  | 3 |  |  | M/2 | 53 | HR | Negative | NA | B/M | CNS, XY | e11;e3 | EME1,HEATR5B,MIB2,NNMT,OLFM1,PTPN11,SRSF2,SURF1 | Alive 6.8y |
|  | 4 |  |  | M/7 | 130.3 | HR | NA | NA | B/M | 47,XY,+X[13]/46,XY[7] | e11;e3 | BBX,C17orf53,DOCK6,FLT3,IL18BP,KLK8,KMT2D,OR8D1,TIMD4,WDR18 | Alive 9.3y |
|  | 5 |  |  | M/9 | 64.6 | HR | CNS1 | NA | B/M | 46,XY,t(2;9)(p21?;p13?),del(19)(p13.3) | e17;e7 | BAZ2B,GLUD1,KRAS,MUC17,NRAS,PCDHGA7,SETD2,UNC80 | Alive 1.7y |
|  | 6 |  |  | F/2 | 11.2 | SR | NA | NA | B/M | NA | e14;e2 | CADM1,FBXW7,PRSS35,PRX,PTPN11,PTPRB | Alive 4.8y |
|  | 7 |  |  | F/6 | 11.3 | SR | CNS2 | NA | B/M | trisomy 8 | e18;e7 | ABCB1,ANP32D,BIRC7,COL5A3,EED,FAM120C,HABP2,LRP1B,MGA,MUC16,MUC19,PNPLA2,SVEP1,TPRX1 | Died 4.5y |
|  | 8 |  |  | M/16 | 29.4 | HR | NA | NA | B/M | NA | e11;e2 | CDKN1B,SET,TCF3 | Alive 3.5y |
|  | 9 |  |  | M/0 | 1500 | HR | positive | NA | B/M | Insufficient metaphase | NA | FLRT2,FLT3,SMAD9 | Died 1.2y |

ALL: acute lymphoblastic leukemia, MPAL: mixed phenotype acute leukemia,AML: acute myeloid leukemia, ALAL:Acute leukaemia of ambiguous lineage, S: sex, A: age, WBC: white blood cell, NCI: National Cancer Institute, CNS: central nervous system, F: female, M: male, HR: high risk, SR: standard risk, NA: not available or not applicable, CR: complete remission, y: year, SCT: stem cell transplantation, OS: overall survival, RFS: relapse free survival
